# Highly Variable Microbiota Development in the Chicken Gastrointestinal Tract

**DOI:** 10.1371/journal.pone.0084290

**Published:** 2013-12-31

**Authors:** Dragana Stanley, Mark S. Geier, Robert J. Hughes, Stuart E. Denman, Robert J. Moore

**Affiliations:** 1 Animal, Food and Health Sciences, Commonwealth Scientific and Industrial Research Organisation, Geelong, Victoria, Australia; 2 Animal, Food and Health Sciences, Commonwealth Scientific and Industrial Research Organisation, St Lucia, Queensland, Australia; 3 Pig and Poultry Production Institute, South Australian Research and Development Institute, Roseworthy, South Australia, Australia; 4 Poultry Cooperative Research Centre, University of New England, Armidale, New South Wales, Australia; 5 School of Animal and Veterinary Sciences, The University of Adelaide, Roseworthy, South Australia, Australia; 6 School of Medical and Applied Sciences, Central Queensland University, Rockhampton, Queensland, Australia; 7 Department of Microbiology, Monash University, Clayton, Australia; Agriculture and Agri-Food Canada, Canada

## Abstract

Studies investigating the role that complex microbiotas associated with animals and humans play in health and wellbeing have been greatly facilitated by advances in DNA sequencing technology. Due to the still relatively high sequencing costs and the expense of establishing and running animal trials and collecting clinical samples, most of the studies reported in the literature are limited to a single trial and relatively small numbers of samples. Results from different laboratories, investigating similar trials and samples, have often produced quite different pictures of microbiota composition. This study investigated batch to batch variations in chicken cecal microbiota across three similar trials, represented by individually analysed samples from 207 birds. Very different microbiota profiles were found across the three flocks. The flocks also differed in the efficiency of nutrient use as indicated by feed conversion ratios. In addition, large variations in the microbiota of birds within a single trial were noted. It is postulated that the large variability in microbiota composition is due, at least in part, to the lack of colonisation of the chicks by maternally derived bacteria. The high hygiene levels maintained in modern commercial hatcheries, although effective in reducing the burden of specific diseases, may have the undesirable effect of causing highly variable bacterial colonization of the gut. Studies in humans and other animals have previously demonstrated large variations in microbiota composition when comparing individuals from different populations and from different environments but this study shows that even under carefully controlled conditions large variations in microbiota composition still occur.

## Introduction

A range of studies have investigated the structure of the microbiome in the gut of broiler chickens. Comparison across studies has proven difficult because of the variety of different methods used; e.g. culture based studies [1], G+C profiling analysis [2], quantitative PCR [3], and 16S rRNA based studies. The 16S-based methods have used a number of different approaches including terminal restriction fragment length polymorphism analysis [4], temporal temperature gradient gel electrophoresis [5], denaturing gradient gel electrophoresis (DGGE) [6], low throughput clone analysis [7] or multivariate curve resolution [8]. More recently high throughput next generation sequence based studies have been performed [9]. Inter-study comparisons are also complicated by the different ways that results have been reported, with some papers detailing populations down to the class and genus level whereas other studies simply demonstrate similarities or differences, for example in DGGE gel profiles, without any detailed quantitative taxonomic information.

Compounding these difficulties are different approaches to sample analysis with some studies looking at results from individual birds and others using pooled samples [10]. The analysis of individual bird samples has demonstrated significant variation in microbiota structure within single treatment groups [11] and the use of pooled samples does not allow the characterization of this potentially important variation. Further complicating any cross-study analysis is the wide variation in experimental or field conditions investigated with variation across the birds (source, breed, age, sex, history), feed, environmental conditions, and different treatments investigated.

Accepting that there are obvious limitations to cross-study comparison, an underlying issue is that there appears to be a high degree of variation in the overall structure of the microbiota observed in different studies. The current study is directed at addressing this finding to determine if the apparent microbiota variation across trials is real or simply an artifact of different experimental designs or analysis methods. This issue is important because the outcome would influence the design of future research into ways in which we might aim to manipulate the microbiota to improve health and productivity. Scientific rigor requires a hypothesis to be supported by replicated results. However, most published studies investigating changes in the microbiota of chickens and other animals have used single trials, usually contrasting the microbiota in two or more treatment groups. In some studies the results of multiple trials with different variables have been reported but only rarely have results been reported for replicated trials. In a previous study we investigated microbiota changes associated with a *Clostridium perfringens* infection and although some key changes in microbiota could be replicated across trials an important finding was that there were large differences in the microbiota in the control groups between trials [12].

We were concerned that even with the carefully controlled conditions applied previously there were significant differences seen from trial to trial. We have now sought to understand and characterize the amount of variation seen in the cecal microbiota of birds across a set of three trials in which the chicken source, feed, and growing conditions were all tightly controlled and replicated as far as practically possible. The results highlight the variability in microbiota structure found across replicate trials and also shows the significant microbiota variation seen between animals within a single uniformly derived and treated group.

## Materials and Methods

### Chicken Trials

The protocol used to perform the animal trials was as described [9] but with a slightly modified feed formula. Briefly, one-day old male Cobb 500 broiler chickens were transferred from a commercial hatchery (Baiada Hatchery, Willaston, SA, Australia) to a rearing pen in a temperature-controlled room. At the hatchery the chicks received the vaccines that are routinely used in broiler chicks in Australia; Marek’s, Newcastle Disease and Infectious Bronchitis. The feed supplied *ad libitum,* comprised of 44.4% of wheat, 17% soybean meal, 15% barley, 10% canola meal, 5% peas, 3.2% meat meal, 3% tallow, 1% limestone, 0.5% vitamin mix, and traces of salt, lysine HCl, DL-methionine and threonine. All of the feed for the replicate trials came from the same batch of commercially prepared crumbles and was stored in cool dry conditions for five months between the first and last trial. The lighting regime for the trials started with 22–23 hours per day gradually reducing to 12 hours per day by day 9 and for the rest of the trial period. For the first 13 days post-hatch the birds were housed together in a single concrete floored pen with fresh, untreated, sawdust and shavings for bedding material. After day 13 the chickens were transferred in pairs to 48 open wire metabolism cages located in a temperature-controlled room (23–25°C). Birds were initially placed in pairs for an acclimation period to minimize stress associated with separation and were then moved into individual cages on day 15. The individual housing prevents competition for feed, minimises behavioural issues and allows for the precise feed intake of each individual chicken to be measured. Birds were culled on day 25 and cecal luminal contents were collected from one ceca from each bird for microbial analysis. Feed Conversion Ratio (FCR) was calculated as a ratio of feed consumed and weight gained. Therefore, lower ratios indicate that the bird is more efficient at converting food into body mass. Three identical trials, trials 1, 2, and 3, were performed over a 5-month period.

### Animal Ethics Statement

All animal work was been conducted according to the national and international guidelines for animal welfare. The animal trials were approved and monitored by the Animal Ethics Committees of the University of Adelaide (Approval No. S-2010-080) and the Department of Primary Industries and Resources, South Australia (Approval No. 08/10).

### DNA Preparation and PCR Amplification of 16S Ribosomal DNA Gene Sequences

DNA was prepared as detailed by Stanley *et al.* [9]. Briefly, total DNA was isolated using the method of Yu and Morrison [13] except that homogenization was done using a Precellys 24 tissue homogenizer (Bertin Technologies) at maximum speed of 6500 rpm, twice, 3 x 10 seconds each time. Quantity and quality of DNA was inspected on a NanoDrop ND-1000 spectrophotometer. DNA was amplified using Bio-Rad iProof DNA polymerase. The primers used amplified the V1–V3 region of the 16S rRNA gene (forward primer [14], 5′ AGAGTTTGATCCTGG 3′; reverse primer, a truncated version of W31 [15], 5′ TTACCGCGGCTGCT 3′) and both primers also incorporated sequences for 454 sequencing. The reverse primers consisted of a set of primers that included a barcode sequence unique to each specific bird sample in a given amplicon pool. Amplification of products was performed in an Eppendorf Mastercycler.

### High Throughput Sequencing and Analysis of 16S rRNA Gene Amplicons

16S rRNA gene amplicons were sequenced using a Roche/454 FLX Genome Sequencer, according to the manufacturer’s instructions. Sff files were split into fasta and qual files using PyroBayes [16] and chimeric sequences removed using pintail [17]. Sequence quality trimming settings were: sequence length 300–600 bases, no ambiguous sequences, minimum average quality score of 25 and maximum homopolymer run of 6 nucleotides, using Qiime v1.3.0 [18]. OTUpipe [19], combining USEARCH and UCLUST scripts [20], [21], was used to perform denoising error-correction, abundance and amplicon estimation and OTU picking. After OTUs were assigned, using 97% sequence similarity, all of the remaining analysis used Qiime v1.3.0 software using Qiime defaults for that version, unless stated otherwise. Taxonomy was assigned using a Blast method against the GreenGenes database [22] and further confirmed using the EzTaxon database [23]. All samples represented by less than 1000 sequences were removed from the analysis. After this step, samples collected from 207 different birds remained; 70, 74 and 63 samples for trials 1, 2, and 3 respectively. Rare OTUs with less than 10 sequences and present in less than 5 samples were removed from further consideration. Normalization of OTU table counts was done by performing multiple rarefactions 100 times and averaging counts. The resulting multiple rarefied OTU table was used for all further analysis including making OTU network tables in Qiime. Networks were visualised in Cytoscape 2.8.0. Significance of between trial differences was inspected using the R package ade4 (**A**nalysis of **D**ata functions for **E**cological and **E**nvironmental data in the framework of **E**uclidean **E**xploratory) [24]. The sequence data and sample metadata have been submitted to the MG-RAST public database under ID No.’s 4537568.3 to 4537776.3.

## Results

### Performance of the Three Flocks

**Figure 1:**
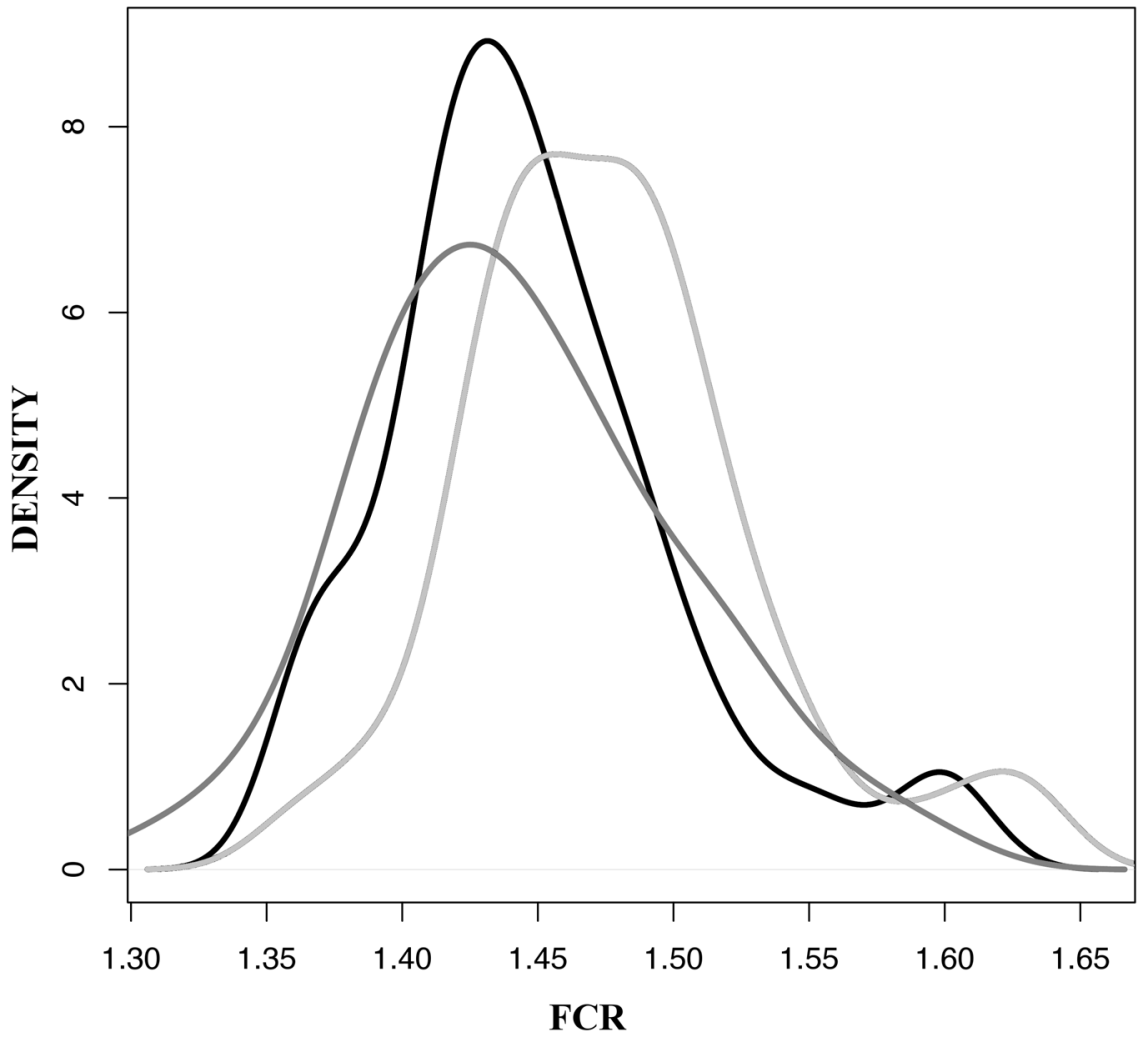
Distribution of FCR values given as a probability density function across the three trials. Trial 1 grey, trial 2 black, and trial 3 light grey.

FCR is the most widely used performance measure in the poultry industry; it represents a measure of how efficiently a bird uses feed towards growth. Since FCR is calculated as the ratio of consumed feed and gained weight, flocks with the lowest FCR values, that need lowest amount of food per kg of weight, are regarded as the best performing. As we measured the FCR of each individual bird we were able to characterize the overall flock performance and build up a detailed profile of the performance of individual birds within each flock (Figure 1). Based on the three distribution plots, it is apparent that the flock from trial 2 had the most desirable FCR distribution. Trial 1 had the lowest mean FCR value, however, trial 2 had a similar mean but a narrower distribution and fewer birds with undesirable extremely high FCR. Trial 3 had the least desirable profile with the highest mean and the highest standard deviation from the mean.

### Cecal Microbiota

A total of 627,613 quality trimmed sequences were produced with an average number of sequences per sample of 2,565, 2,899 and 3,705 for trials 1, 2 and 3, respectively. ANOVA (p-value <0.05) was used as a statistical measure to define OTUs that were in differential abundance between trials. This showed that 58% of all OTUs were differentially abundant. Reducing the p-value to 0.001 resulted in 30.3% of all OTUs being identified as differentially abundant between the three trials. The lowest Bonferoni corrected Qiime ANOVA p-value was 2.47e^−27^, indicating that the differences were highly statistically significant.

**Figure 2:**
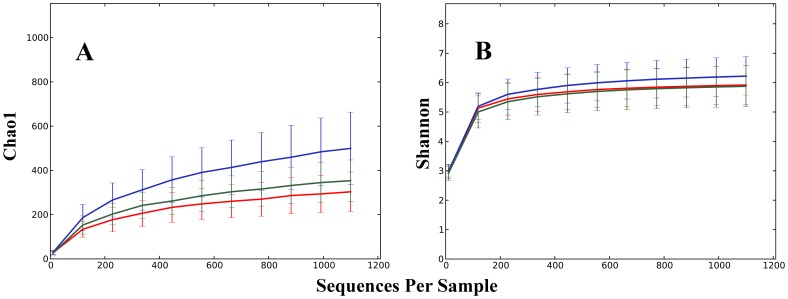
Alpha diversity plots across the three trials. Rarefaction plots for samples from trial 1 (red), trial 2 (green) and trial 3 (blue), based on Chao1 (A) and Shannon index (B).

**Figure 3:**
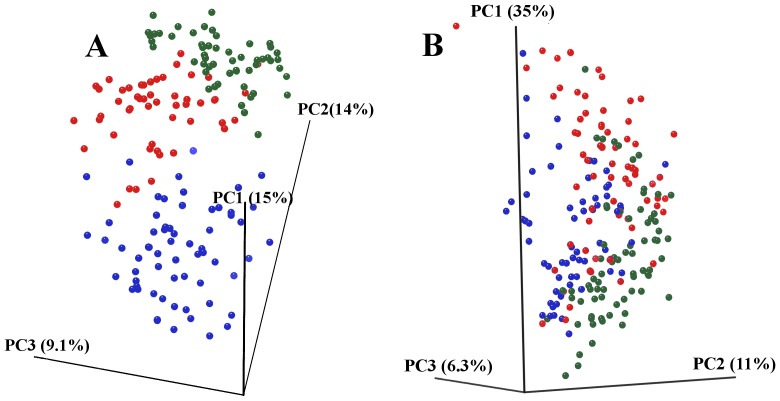
Beta diversity plots across the three trials. 3D PCoA plots based on unweighted (A) and weighted (B) UniFrac from trial 1 (red), trial 2 (green) and trial 3 (blue).

**Figure 4:**
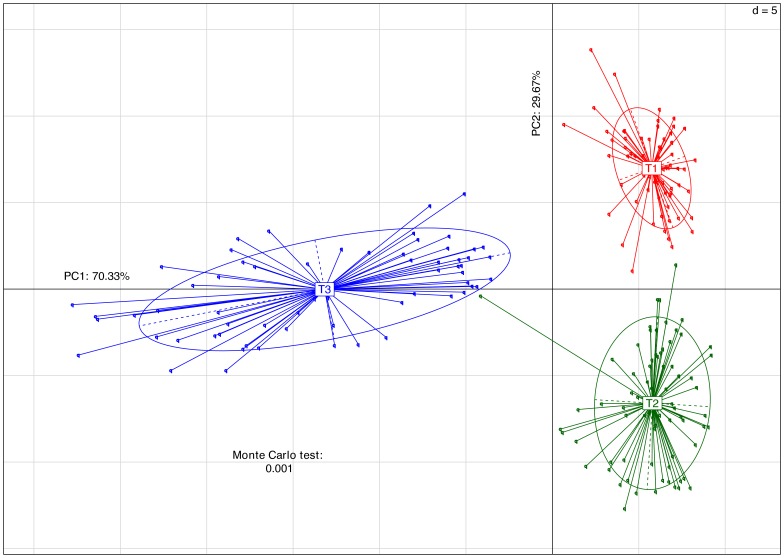
Multivariate analysis PCA plot. The plot is based on between groups (trials) analysis using the ade4 R phylogenetic package. Monte Carlo testing was applied using 999 permutations.

**Figure 5:**
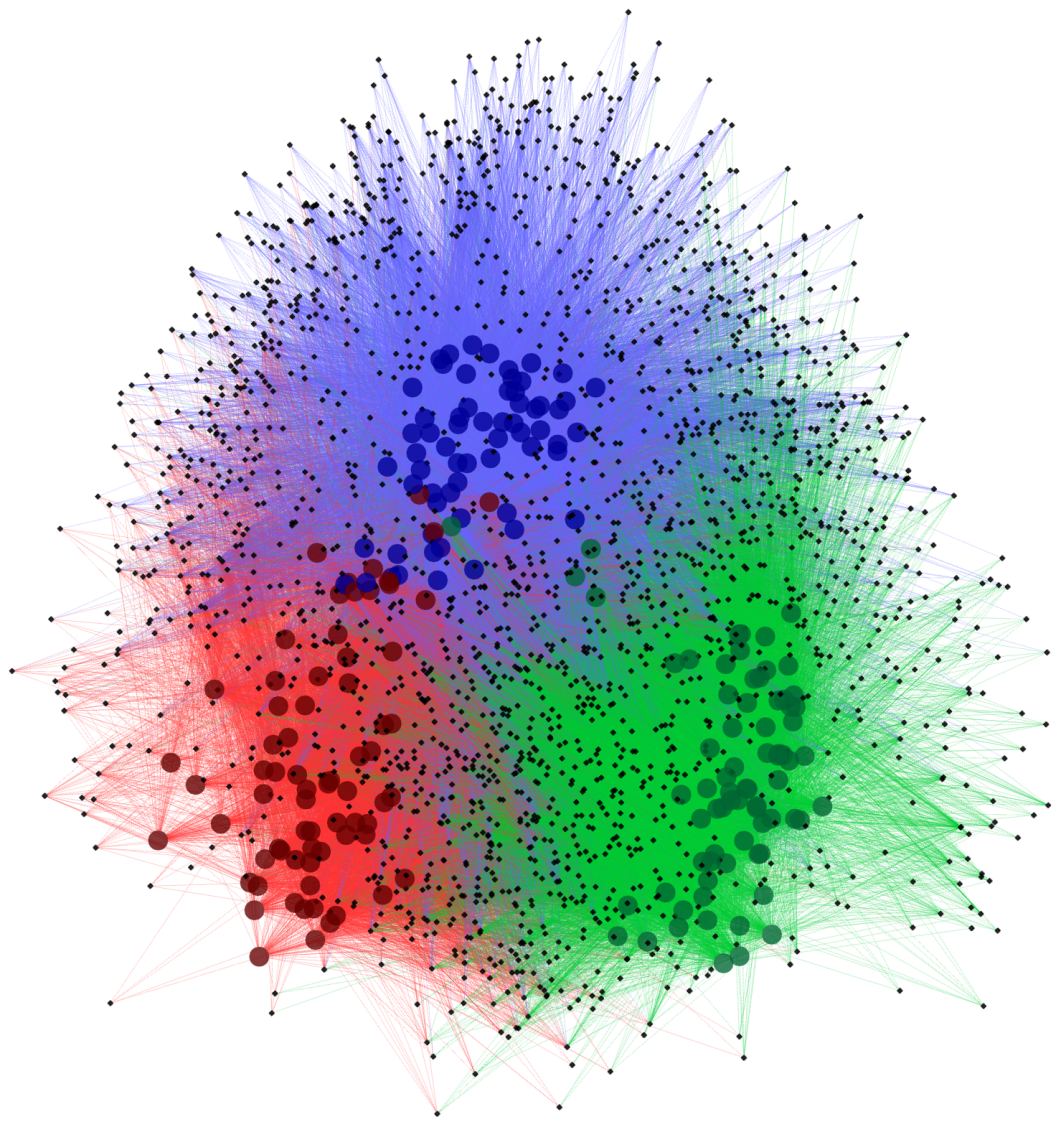
OTU network map. The network represents OTU interactions between multiple rarified samples originating from trial 1 (red), trial 2 (green) and trial 3 (blue) and OTUs (black). Edges (lines) join a sample and an OTU present in that sample and are colored according to sample are derived from.

**Figure 6:**
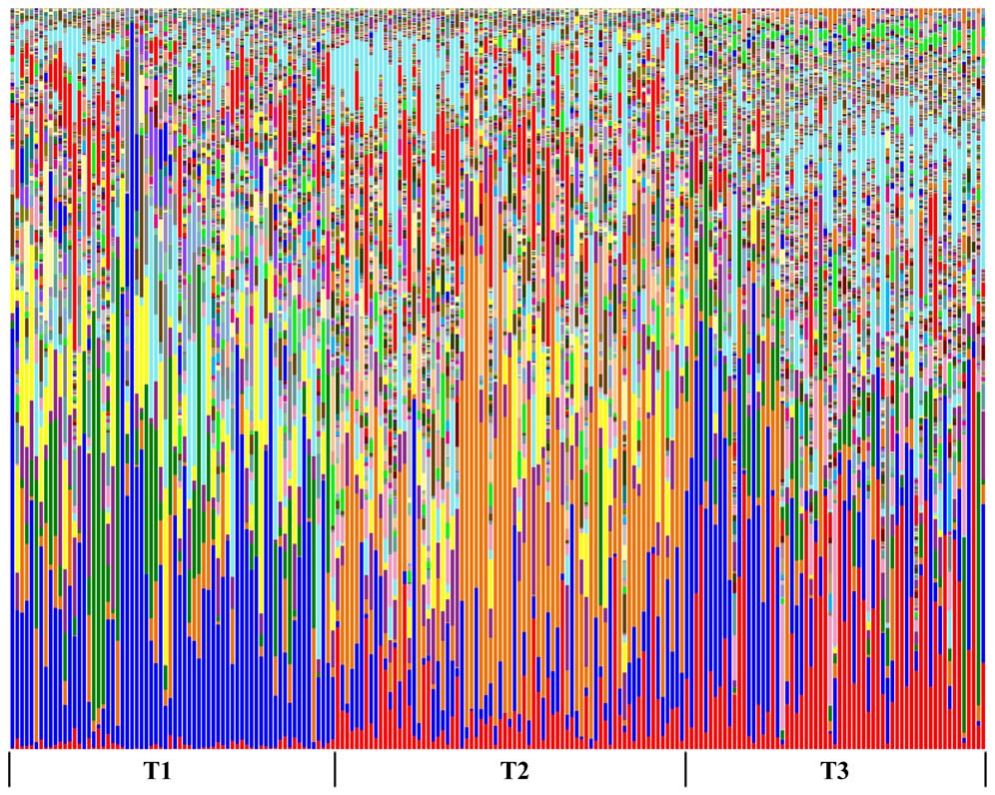
Bar chart showing distribution of OTUs at 5% divergence. Each individual OTU is represented with a different colour. The chart demonstrates differences in microbial profiles between the trials.

Alpha and beta diversity were inspected using all the metrics available in the Qiime package. Alpha diversity metrics showing significant difference between trials were: Chao1 (Figure 2A), Observed Species, PD Whole Tree and Singles with all curves resembling the plot given in Figure 2A. The alpha diversity metrics show the difference in the number of OTUs at chosen phylogenetic levels. Each of the metrics demonstrated that trial 3 had the highest number of species and, based on the Singles alpha metric, also the highest number of low abundance OTUs. There were only slight differences in alpha diversity identified using Simpson, Reciprocal Simpson, Shannon (Figure 2B), Dominance, Doubles or Equitability protocols. This group of alpha diversity metrics reveal the distribution of OTU abundance to determine if a few species dominate or there is more equal species distribution. Based on these results all samples from the three trials had similar OTU distribution with no strongly dominant taxa. Non-phylogenetic beta diversity metrics grouped samples from the three trials into three fully separated groups; the Spearman metric completely separated samples from trial 3 from two separated but close groups of samples originating from trials 1 and 2. Unweighted (Figure 3A) and Weighted (Figure 3B) Unifrac also showed some but not total separation of the samples from each trial. Between trials PCA analysis (Figure 4) performed in the R ade4 phylogenetic package, demonstrated that the microbiota structure of the birds in each trial are different with Monte Carlo p-value of 0.001. Based on the PCA component loadings, the PC1 axis is most influenced by *Lactobacillus crispatus* and *Lactobacillus helveticus* and in the opposite direction *Lactobacillus reuteri*. PCA2 is most determined by *Parabacteroides distasonis, Lactobacillus taiwanensis* and an unknown *Clostridiales* in one direction and *Bacteroides fragilis* in the opposite direction. Generally, OTUs driving the difference between the trials were *Lactobacillus*, *Clostridium* and *Bacteroides*-related as demonstrated in OTU bar-plots provided in Figure S1. This is further emphasized by the weighted network diagram (Figure 5) which not only shows the separation of birds across trials but also confirms that trial 3 had much more diverse samples, many elements of which are not shared with the birds in trials 1 and 2. The differences at an OTU level can be clearly seen in the bar chart (Figure 6). Trial 3 birds had higher bird to bird variation and more diverse microbiota. Even at a high taxonomic level there were differences between the trials; for example, the ratio of Bacteroidetes to Firmicutes was 0.41, 0.13 and 0.22 for trials 1, 2, and 3 respectively, indicating large differences in the microbiota of each trial.

## Discussion

This study has, for the first time, analysed the cecal microbiota of a large number of individual birds distributed across a series of repeated trials conducted over a 5-month period. The three replicate trials used healthy birds, sourced from the same hatchery, fed the same food and reared under similar conditions, to investigate batch effects from one trial to another. Access to such a large data set, across multiple replicated trials and many birds, has allowed us to make some interesting comparative observations about the structure of chicken gut microbiota across and within trials. The data presented shows unexpectedly high differences in the microbiota between the three trials. Clearly there is a high degree of variability in the establishment and maintenance of the microbiota and this may influence the health and productivity of birds as suggested by the different FCR performance profiles seen for each flock. Studies in humans [25] and other animals [26] have previously demonstrated that despite the possible presence of a core microbiota there are large variations in overall microbiota composition when comparing individuals from different populations and from different environments but the current study shows that even under carefully controlled conditions large variations in microbiota composition can occur.

The advances in technology for microbiota analysis have resulted in a flood of new studies linking microbiota to various health outcomes. Intestinal microbiota has recently been identified as a major determinant of health and wellbeing in experimental animal model systems and in humans. The microbiota can influence the host in a range of different ways including (i) providing a source of digestive enzymes and thus enhancing nutrient availability, (ii) outcompeting and destroying potential pathogens and (iii) ensuring development of a healthy immune system [27]. Aberrant microbiota development, resulting in alterations of intestinal microbial colonisation have been associated with allergies, obesity, diabetes and altered immune system development [27]. Differences in intestinal microbiota between batches of newly hatched chickens may account for different responses to antibiotic treatment and different susceptibility to disease [28], [29]. Thus, across the animal kingdom, development of a healthy intestinal microbiota is a crucial step in the first days of life that may determine future health and fitness.

Because of the potential health and productivity influences of the gut microbiota it is important to consider the possible causes of the high level of variation seen across replicated trials and how this variation could be controlled and reduced. In humans it has been reported that deprivation of maternal microbiota during caesarean birth produces aberrant microbiota in infants that is more representative of microbiota derived from skin and environmental bacteria rather than vaginally derived bacteria [30]. The spread of fecal bacteria is diminishing in modern infants. For example, establishment of common fecal bacteria is delayed in western countries, while in countries in the middle east and Africa even caesarean section delivered infants acquire stable fecal populations within a week [31]. Adlerberth *et al* [31] suggested that excessive hygiene limits circulation of fecal bacteria today, thus altering microbiota composition in newborns. The same authors suggested that the recorded increase, over the last decade, in Staphylococci, a bacterium not previously regarded as intestinal, occurs due to lack of competition from “professional” gut microbiota. The fact that colonization with microbiota previously foreign to the gut is facilitated by standard hygienic measures may cause some concern. We speculate that we may be seeing a somewhat similar effect in the trials reported here, with the chickens randomly colonised by bacteria originating from the wider environment rather than predominantly from maternally derived bacteria.

Arriving into the world by hatching from an egg is very different to mammalian birth but they share in common the almost immediate exposure of the young to maternal microbiota. In mammals the newborn are immediately exposed to vaginal bacteria during birth. In birds the hatching chick is exposed to bacteria on the surface of the egg and in the immediate nest environment; the bacteria on the egg surface and in the nest are largely derived from the parents. However, in modern commercial chicken hatcheries high hygiene levels are generally maintained; eggs are usually washed and fumigated to remove bacterial contamination, and hence chicks are not exposed to the same bacteria that they would likely see in a natural setting. Rather, they must be colonized by bacteria encountered in the wider environment, for example in the boxes that they are transported in, from the first feed that they receive, and from the people handling them. We hypothesize that this somewhat random exposure to bacteria, from a variety of different sources, is the basis for the wide batch to batch variation seen in the structure of gut microbiota that we have observed in the trials reported here.

Apajalathi *et al.* [2] have demonstrated that the first days of a chicks life are critical in establishing a normal stable microbiota [2]. This indicates that young chicks are often well advanced in establishing a stable microbiota before they leave the hatchery. A future goal, which was beyond the scope of this study, is to determine whether microbiota variability in chicks can be controlled by deliberate exposure to adult microbiota.

The high cost of both animal trials and sequencing has resulted in most published microbiota studies in different experimental and production animals originating from a single trials. The data present here strongly point to problems in reproducing this kind of data even when using near identical conditions. Moreover, the high within batch animal to animal variation suggests a need for high numbers of samples if the microbial profile investigated is to be faithfully represented, even within a trial. These considerations should be taken into account when designing experiments to study animal microbiota.

## Supporting Information

Figure S1 **Boxplots of the OTUs most differentially abundant (p<10^−10^) between the 3 trials.** Generated using R phylogenetic package ade4 and Qiime analysis outputs. The p-values are calculated using Qiime ANOVA. For OTUs with similarity to closest type strain in EzTaxon database >95%, taxonomy is given as EzTaxon strain and similarity, for OTUs with lower similarity taxonomy is given at an order level.
(DOCX)
