# Supplementary material for: Highly Variable Microbiota Development in the Chicken Gastrointestinal Tract
Source: PLoS One. 2013 Dec 31;8(12):e84290. doi: 10.1371/journal.pone.0084290 (PMC3877270; doi:10.1371/journal.pone.0084290)
Supplement: Figure S1 — Boxplots of the OTUs most differentially abundant (p<10−10) between the 3 trials. Generated using R phylogenetic package ade4 and Qiime analysis outputs. The p-values are calculated using Qiime ANOVA. For OTUs with similarity to closest type strain in EzTaxon database >95%, taxonomy is given as EzTaxon strain and similarity, for OTUs with lower similarity taxonomy is given at an order level. (DOCX) [file pone.0084290.s001.docx]

**Highly variable microbiota development in the chicken gastrointestinal tract: are hygiene levels to blame?**

Dragana Stanley^1,4,6^, Mark S. Geier^3,4,5^, Robert J. Hughes^3,4,5^, Stuart Denman^2^ and Robert J. Moore^1,4,7,*^

**Supplementary Figure**


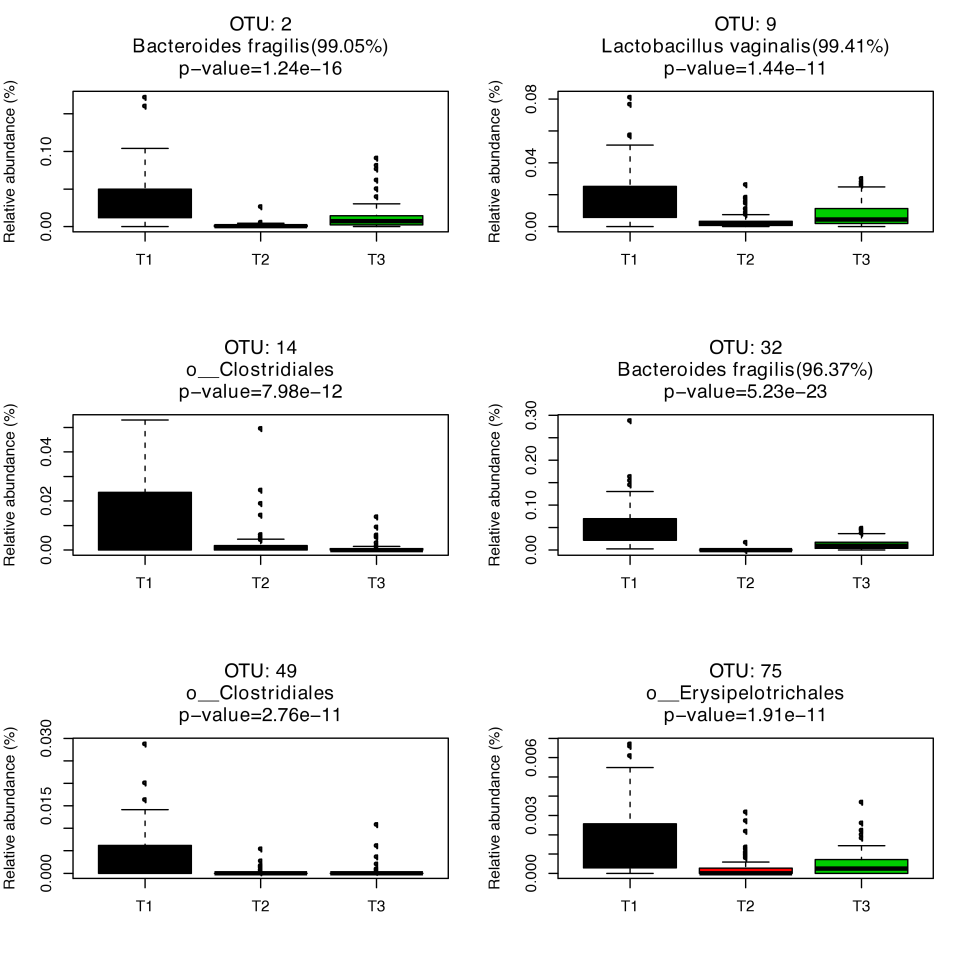

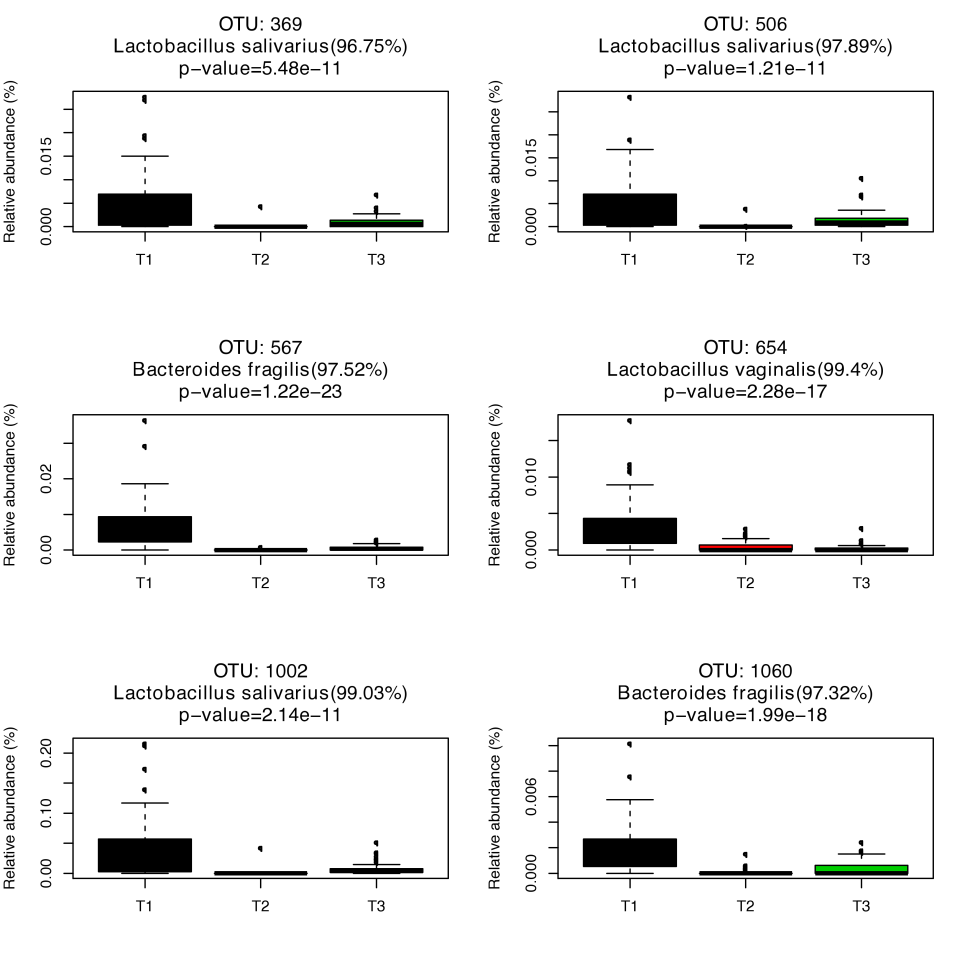

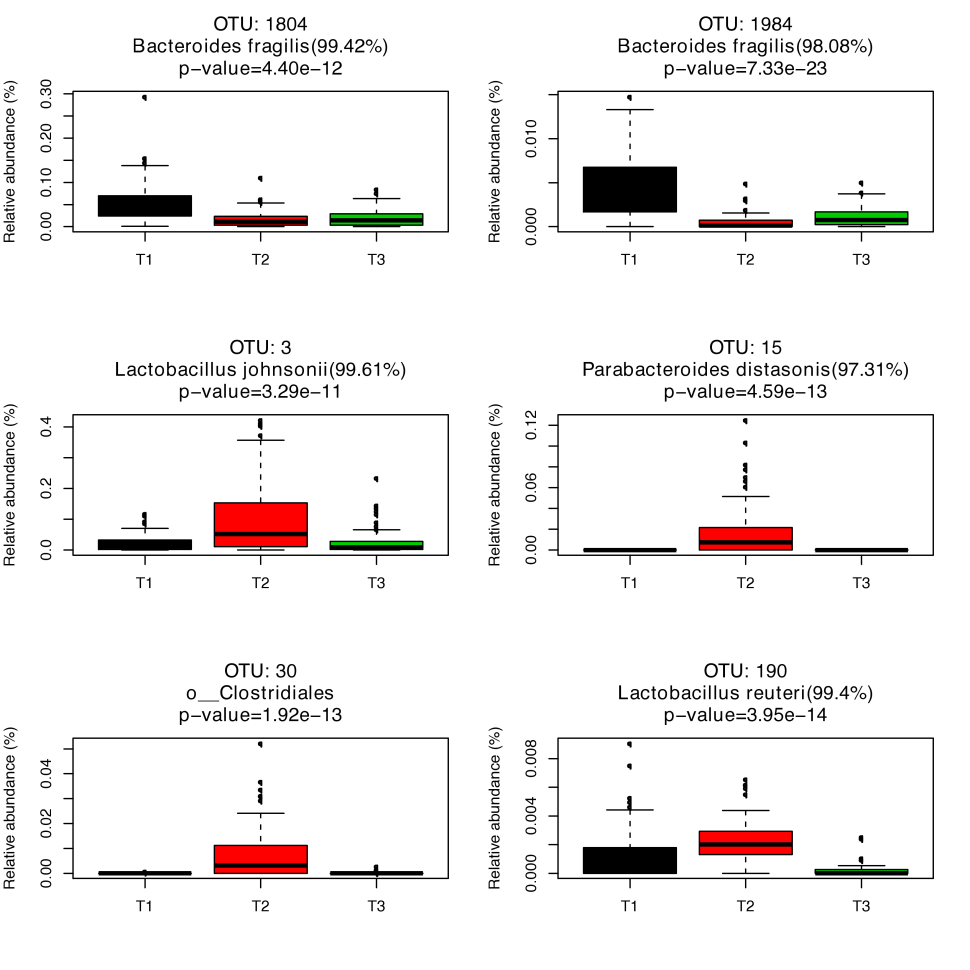

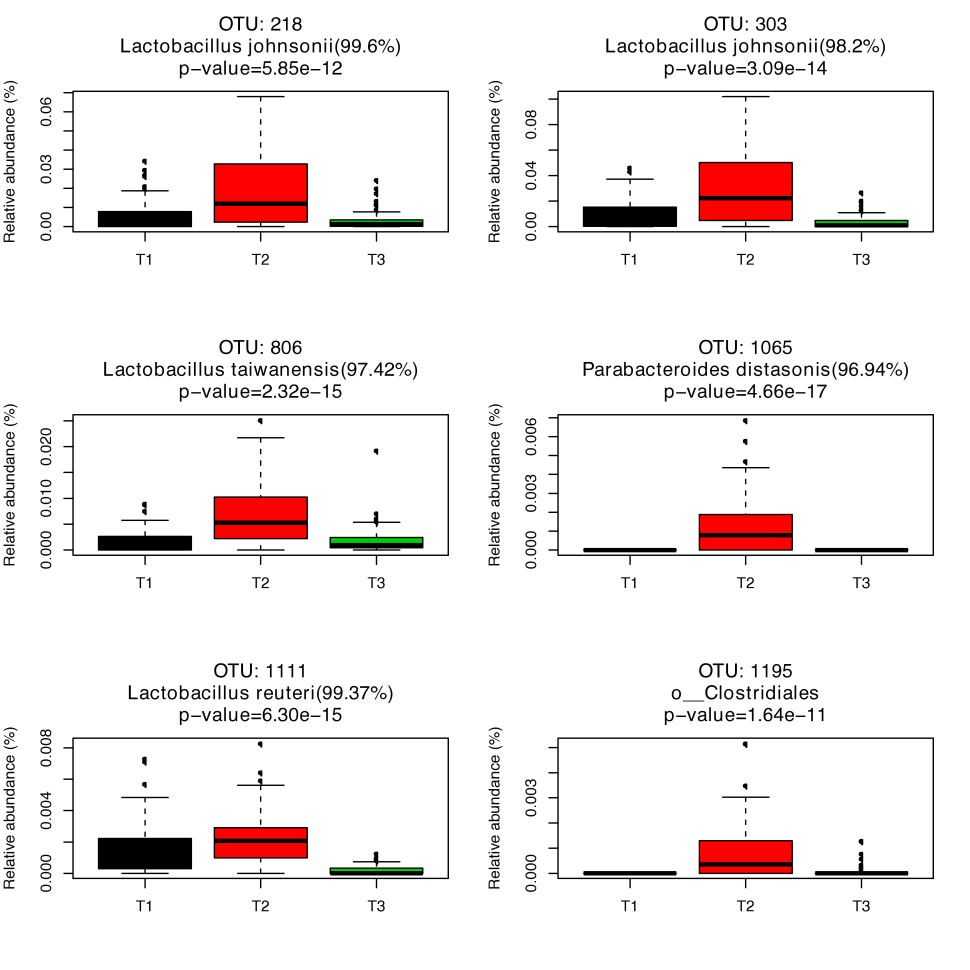


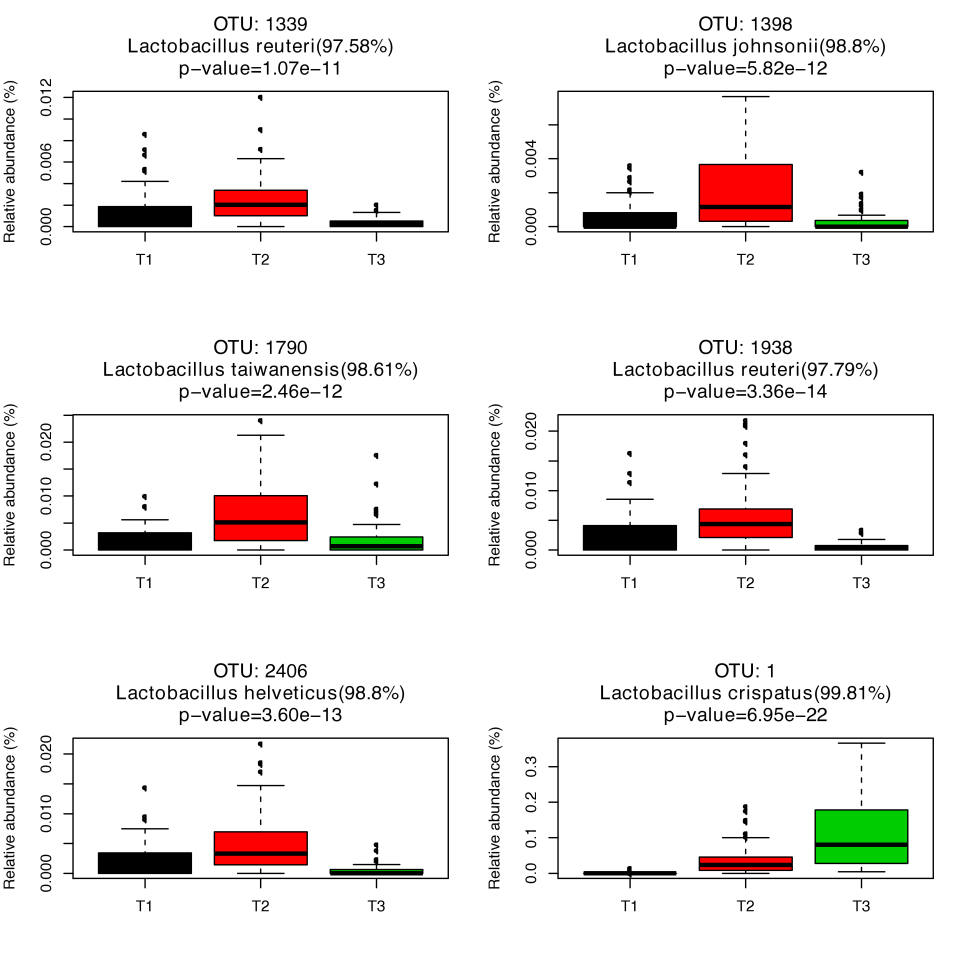

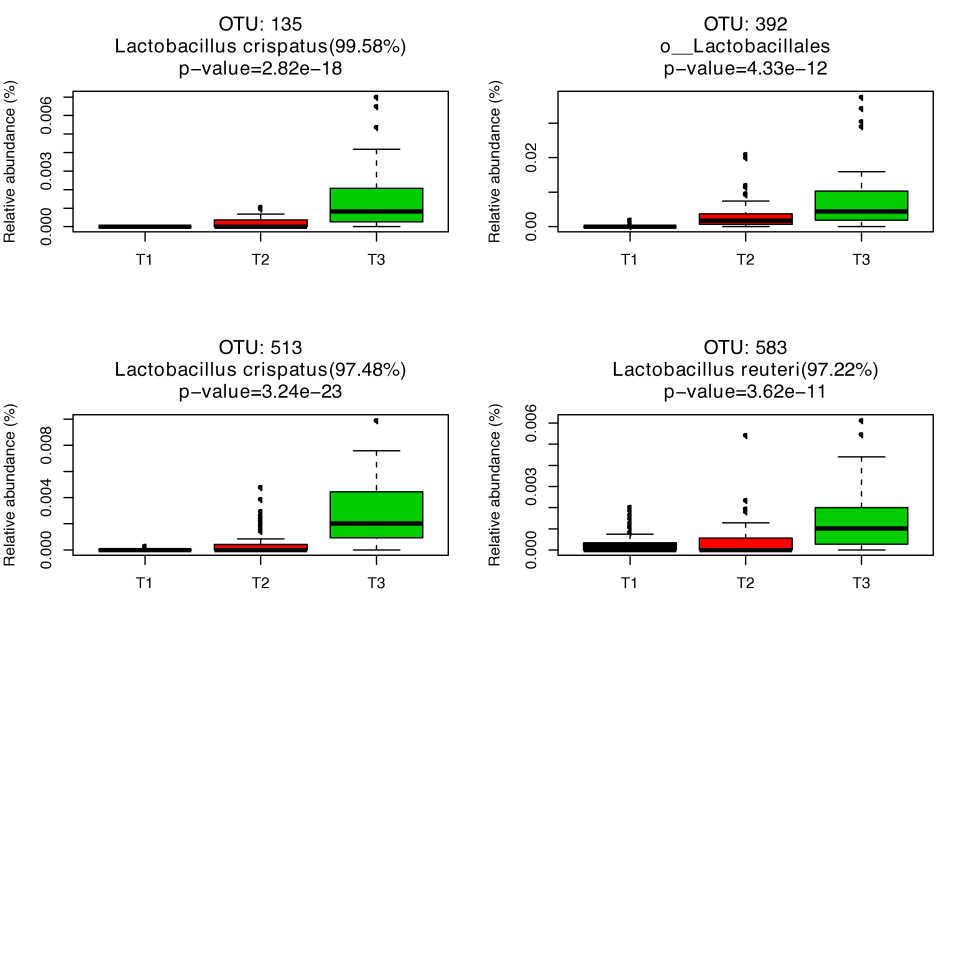


**Figure S1:** Boxplots of the OTUs most differentially abundant (p<10^-10^) between the 3 trials, generated using R phylogenetic package ade4 and Qiime analysis outputs. The p-values are calculated using Qiime ANOVA. For OTUs with similarity to closest type strain in EzTaxon database > 95%, taxonomy is given as EzTaxon strain and similarity, for OTUs with lower similarity taxonomy is given at an order level.
